# In Vitro Chronic Hyperinsulinemia Induces Remodelling of Vascular Smooth Muscle Cells from Young Men and Women in a Sex Hormone Independent Manner

**DOI:** 10.3390/pathophysiology32010012

**Published:** 2025-03-13

**Authors:** Ashley Jazzar, Danielle Jacques, Amira Abou-Aichi, Ghassan Bkaily

**Affiliations:** Department of Immunology and Cell Biology, Faculty of Medicine and Health Sciences, University of Sherbrooke, Sherbrooke, QC J1H 5N4, Canada; ashley.jazzar@usherbrooke.ca (A.J.); amira.abou-aichi@usherbrooke.ca (A.A.-A.)

**Keywords:** human vascular smooth muscle cells, insulin, hyperinsulinemia, sex dependency, sex hormones, calcium overload, ROS, glutathione, vascular hypertrophy

## Abstract

Elevated circulating insulin levels between 80 and 100 µU/mL characterize hyperinsulinemia, which often leads to metabolic disorders such as obesity, insulin resistance, and type 2 diabetes (T2D). Elevated circulating insulin levels can directly affect vascular function and contribute to the pathophysiology of the cardiovascular system, including secondary arterial hypertension (SAH) and atherosclerosis. It is well known that hyperinsulinemia induced remodeling of the heart. However, there is no information on whether intrinsic differences exist between human vascular smooth muscle cells (VSMCs) and if in vitro mimicking hyperinsulinemia induces human VSMCs morphological and intracellular homeostasis remodeling in a sex- and sex hormones-dependent manner. Our in vitro cultured human VSMCs, coupled with quantitative 3D confocal imaging results, show that intrinsic differences exist between VSMCs from young men and women. Chronic hyperinsulinemia (80 µU/mL, 48 h treatment) increases cell and nuclear volumes associated with increased intracellular calcium (Ca^2+^) and ROS and decreased glutathione. In the absence of hyperinsulinemia, pretreatment with testosterone in VSMCs from men and oestradiol in VSMCs from women had no effect. Both sex hormones partially but not completely prevented hyperinsulinemia-induced remodeling of VSMCs from young men and women. The increase in VSMC volume may increase the thickness of the tunica media, leading to a decrease in the lumen of the blood vessel, which promotes the development of SAH and atherosclerosis in a sex-dependent manner.

## 1. Introduction

There is no precise and universally accepted definition of hyperinsulinemia [1,2]. However, it is well-accepted that hyperinsulinemia refers to an elevated blood insulin level, often seen in conditions like insulin resistance or metabolic syndrome [2]. Previously, insulin resistance was considered the primary etiological factor in the development of obesity, type 2 diabetes (T2D), and cardiovascular diseases [2,3]. In addition, compensatory hyperinsulinemia was thought to be a direct consequence of insulin resistance [2,3]. Recently, a new hypothesis has emerged that chronic hyperinsulinemia precedes and leads to insulin resistance, obesity, and T2D [2,4]. Furthermore, hyperinsulinemia can occur in the presence or absence of hyperglycemia, such as during in utero development, and in children and adolescents [3,4,5].

Many studies were done on hyperinsulinemia in blood vessels, particularly in cultured vascular endothelial cells and VSMCs of different animal species [6], which are affected by high circulating insulin [7]. The latter can have various effects on contractile VSMCs [7,8]. The remodeling of VSMCs under hyperinsulinemic conditions is a significant factor in the development of cardiovascular diseases, as this remodeling can contribute to vascular dysfunction, diabetes, SAH, stiffening of the arteries, vascular resistance, and atherosclerosis [4,6,7,8]. Intracellular Ca^2+^ plays a key role in regulating the contraction and function of contractile VSMCs [7,8]. In normal conditions, the Ca^2+^ influx into these cells helps regulate their contraction, maintaining vascular tone and blood pressure [7,8,9]. However, hyperinsulinemia’s relationship between chronic high insulin and intracellular Ca^2+^ homeostasis is dysregulated [10,11].

Insulin can directly affect VSMCs through its receptor, activating various signaling pathways such as the PI3K-Akt and MAPK pathways [4,6,12]. These pathways can influence cell proliferation, migration, and survival, contributing to the remodeling of the vasculature [2,4,6,7,13]. Over time, this remodeling can increase vascular resistance and contribute to the development of SAH and atherosclerosis [4]. Contractile VSMCs remodeling can also be impaired/exacerbated by hyperinsulinemia-induced vascular endothelial cell dysfunction by releasing vasoactive compounds such as endothelin-1 (ET-1) and nitric oxide (NO) [6,8]. Hyperinsulinemia, which leads to insulin resistance, can disrupt the balance between vascular endothelium vasoconstrictor ET-1 and vasodilating NO, contributing to an environment that favors VSMCs hypertrophy [6,7,8]. Insulin was reported to induce activation of the R-type Ca^2+^ channel and Ca^2+^ release from the sarcoplasmic/endoplasmic reticulum (SR/ER) [14]. In addition, hyperinsulinemia has been shown to alter Ca^2+^ handling in VSMCs [14]. The dysregulation of cytoplasmic and nuclear Ca^2+^ homeostasis can lead to intracellular Ca^2+^ overload, affecting VSMCs contraction and cell growth. Abnormal intracellular Ca^2+^ levels can also promote VSMC hypertrophy, which may lead to the thickening of the tunica media and contribute to the development of hypertension and atherosclerotic plaques [7,8]. Whether hyperinsulinemia promotes VSMC hypertrophy in young men and women needs to be verified.

The interaction between hyperinsulinemia, VSMCs, and intracellular Ca^2+^ is essential in vascular pathophysiology [4,7,8]. It could be a therapeutic target for treating insulin resistance (where the body’s cells become less responsive to insulin) and associated cardiovascular diseases such as metabolic disorders like T2D, obesity, and metabolic syndrome [4,6]. Hyperinsulinemia can lead to increased levels of circulating triglycerides and very low-density lipoproteins (VLDL), which are linked to the development of atherosclerotic plaques [6,15,16]. Hyperinsulinemia is linked to increased cardiac hypertrophy through insulin-like growth factor (IGF-1) signaling pathways, increasing the size of individual cardiac muscle cells [11]. Whether the latter occurs at the level of human VSMCs is still yet to be discovered.

Sex differences are not always considered in fundamental research, clinical studies, or developing prevention strategies and therapeutic targets [17,18]. Despite the well-documented sex differences in numerous cardiovascular diseases, it was not until recently that women were included in most clinical trials [18]. It is well accepted that essential arterial hypertension (EAH) as well as SAH, such as in hyperinsulinemia and T2D are higher in young men compared to young women [18,19,20]. However, in aging men and women (menopausal), the rate of T2D and SAH becomes similar in both sexes [21,22]. This could be due to the decrease in sexual hormones in menopausal women. In addition, sexual hormones in men and women were reported to have antioxidant properties [23]. These properties and levels will affect different cell functions in men and women [23,24,25]. However, all of this reported work was done in vivo studies, and no work was done in vitro human conditions to verify whether these differences occur at the cell level, such as in VSMCs from young men and women.

Since it is impossible to study the effect of hyperinsulinemia in VSMCs in vivo human clinical trials, it is possible to do such work in vitro or ex vivo in our research group due to the availability of isolated cultured VSMCs from different men and women organ donors. Thus, in this study, we used isolated VSMCs from clinically healthy young men and women donors (supplied by Quebec Transplant) as well as the quantitative 3D confocal to measure the volume (expressed in μm^3^) of the cell and the fluorescence intensity (expressed by μm^3^) of the nuclear and cytosolic regions separately. In the present study, we verified the hypothesis that fundamental intrinsic differences exist between isolated and cultured VSMCs from young men and women and that chronic high levels of insulin-mimicking hyperinsulinemia induce remodeling of human VSMCs independent of sex hormones.

## 2. Materials and Methods

### 2.1. Isolation and Culturing of Human Vascular Smooth Muscle Cells

The procedure for isolating and culturing hVSMCs was described previously [26,27]. The procedures were done in compliance with the institutional review committee’s requirements for using human tissues of Caucasian donors and the consent that the organ donors (for transplantation) give to Quebec Transplant. In brief, the aortas were dissected by the Quebec Transplant surgeon and transported to our laboratory for immediate cleaning and to ensure they were healthy. hVSMCs were isolated from the aortas of young men (16–33 years old) and women (18–26 years old) clinically declared healthy organ donors. The aorta was opened and cleaned from blood. For isolating hVSMCs, the aorta was exposed to trypsin, then washed with M199 solution containing 5% fetal bovine serum (*v*/*v*). The endothelial cells were removed, and the hVSMCs were gently isolated from the first and last layers of the tunica media using a scalpel. This guarantees that these are contractile VSMCs, since non-contractile cells are present in the middle of the tunica media [7]. The cells were centrifuged for 10 min at 200 g and resuspended in the culture medium. As reported previously, isolated hVSMCs were cultured in Petri dishes [26,27]. At confluence, hVSMCs were again isolated and recultured on glass coverslips placed in the culture dishes [26,27]. The purity of cultured hVSMCs was verified morphologically by being elongated cells and spindle shape as classically described and using immunofluorescence coupled with antibodies directed against contractile proteins (myosin light chain kinase, anti-myosin light chain kinase/MLCK antibody, Abcam, Waltham, MA, USA; SM α-actin, anti-alpha smooth muscle actin antibody, Abcam, USA; SM MHC, anti-smooth muscle myosin heavy chain 11 antibody, Abcam, USA; and SM22a, anti-TAGLN/transgelin antibody, Abcam, USA). We also used pharmacological tools of ion channels and transporters (VOCCs, nifedipine; K+ channels, TEA; NCX, NHE, etc.) against the increase of intracellular Ca^2+^ as well as signaling molecules required for the cells contraction that are specific to the contractile phenotype [7].

### 2.2. Pretreatment with High Insulin Levels and Sexual Hormones

The method used for high insulin levels and sexual hormone treatment was similar to the one reported on cardiomyocytes [11]. In brief, for long-term treatment, after two days in culture, the VSMCs from young men and women were treated for 48 h in the absence and presence of insulin (80 μU/mL), insulin (80 μU/mL) + sex hormones (10^−9^ M testosterone (Sigma-Aldrich, St. Louis, MO, USA) and 10^−10^ M 17β-Estradiol (Sigma-Aldrich, St. Louis, MO, USA)) and sex hormone alone (10^−9^ M testosterone (Sigma-Aldrich, St. Louis, MO, USA) and 10^−10^ M 17β-Estradiol (Sigma-Aldrich, St. Louis, MO, USA)). In addition, the concentration of 80 μU/mL was selected as it is similar to the insulin levels observed in hyperinsulinemia, usually between 80–100 μU/mL. It was used after testing several insulin concentrations against cytosolic and nuclear Ca^2+^ [14]. The sex hormone concentrations were similar to normal levels of hormones in young men (testosterone) and women (estrogen).

### 2.3. Quantitve 3D Confocal Microscopy

As previously described, hVSMCs were studied using quantitative 3D confocal microscopy of a Bio-Rad system [26]. In summary, the laser line (9.0 mV) was directed to the hVSMCs and was filtered with a 1–3% neutral density filter to prevent photobleaching of the fluorescent dye. The confocal settings (pinhole size, image size, pixel size, laser line intensity, photometric gain, photomultiplier tube settings, and filter attenuation) were kept unchanged in all the image recordings. The size between the cell sections (16–20 sections/cell depending on the value of the z line) was kept near zero to construct the real image of the cell in 3D. As reported previously, the nucleus was labeled with the fluorescent probe of nucleic acids, syto-11 (Molecular Probes) [26]. Real 3D images were analyzed, and the fluorescence level was quantified and expressed in μm^3^ using an ImageSpace V3.20 analyzing system. This program allows the generation of quantitative 3D images by measuring the volume of the cell (expressed in μm^3^).

### 2.4. Determination of the Cell Volume

Recorded images were transferred to an analysis station of Silicon Graphics equipped with ImageSpace 3D analysis and reconstruction software from Molecular Dynamics [26]. Images of cell volume and Ca^2+^ measurements were performed on real three-dimensional reconstructions. The nucleus region, marked with syto 11, was isolated from the rest of the cell by lowering the intensity threshold to delineate the pixels in this space. This method enabled us to create a true three-dimensional reconstruction of the nucleus alone or the cell without the nucleus. This method allowed us to measure the fluorescence intensity values of the volume of the cell and the Fluo-4/Ca^2+^ complex of the nuclear region and the cytosolic region separately, eliminating any contribution from the other compartment. By isolating the nucleus from the surrounding cytosolic region, it is possible to measure the intensity values of the nuclear volume, eliminating any possible contribution from perinuclear space [26].

### 2.5. Loading with the Calcium, Glutathione, and ROS Fluorescent Probes

The cell membrane permeable Ca^2+^ fluorescence dye Fluo-4/AM (Molecular Probes, Eugene, OR, USA) was used to load Fluo-4 into the cytoplasm and the nucleoplasm of hVSMCs, as reported in our previous published work [26]. The Ca^2+^ fluorescent probe Fluo-4 is homogeneously present in the cytosol and the nucleus of hVSMCs [26] and can be expressed in free Ca^2+^ concentration using a calibration method described previously [26]. For ROS imaging studies, cells were loaded respectively with the ROS probe, 6-carboxy-2′,7′-dichlorodihydrofluorescein diacetate (carboxy-H2DCF-DA; Life Technologies, Burlington, MA, USA), and cell tracker CMFDA (Molecular Probes, Eugene, OR, USA) fluorescent probe to measure intracellular glutathione. These methods have been described and developed previously [26].

### 2.6. Statistical Analyses

As reported previously [26], this work expresses intracellular Ca^2+^, ROS, and glutathione levels as means ± SEM. n represents the number of hVSMCs from a minimum of three different experiments. N is the number of human donors. Results are compared to control values unless indicated differently. Statistical significance was determined using one-way and two-way repeated measure ANOVA (*p* < 0.05) using the Bonferroni post hoc method where applicable for data comparison and analysis by the program Graph Pad Prism 8.

## 3. Results

### 3.1. Effect of a 48 h Treatment of Hyperinsulinemia (80 μU/mL) on the Volume of VSMCs from Men and Women

**Figure 1:**
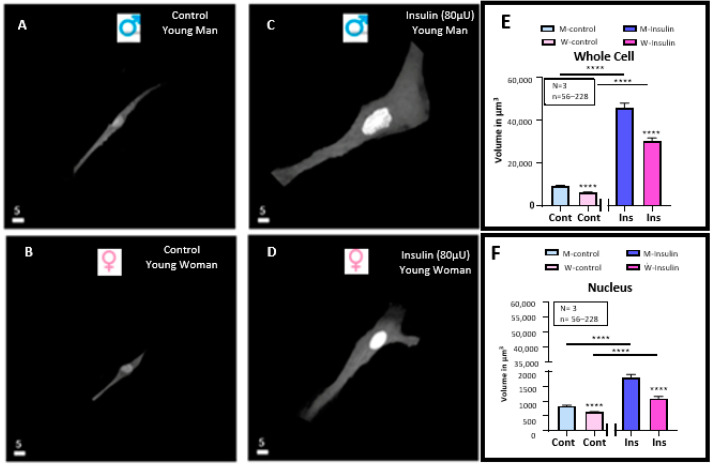
Effect of a 48 h treatment of hyperinsulinemia (80 μU/mL) on the volume of VSMCs from men and women. Examples of a typical quantitative 3D top-view image of VSMCs: in absence of hyperinsulinemia in VSMCs from men (**A**) and women (**B**) and presence of hyperinsulinemia in VSMCs from men (**C**) and women (**D**) with statistical analysis of their whole cell (**E**) and nuclear (**F**) volumes. Panels (**E**,**F**) show a significant increase in cell and nuclear volumes of VSMCs from both men and women induced by 48 h of hyperinsulinemia. Nuclei are labeled with syto-11. White scale bar is in μm. Values are expressed in μm^3^ and represented as mean ± standard error of the mean. N indicates number of donors, while n refers to number of cells. **** *p* < 0.0001. M: men, W: women, Cont: control, Ins: insulin.

In the first series of experiments, we wanted to verify whether VSMCs and their nuclear volume differ in men and women, using quantitative 3D confocal microscopy coupled with the nuclear probe syto-11. In this series of experiments, VSMCs isolated from young donors were used as described in the experimental protocol in the materials and methods section. Figure 1A,B shows a typical example of the cells, and Figure 1E,F summarizes the results. As can be seen in Figure 1A,B, the apparent VSMC volume of men (Figure 1A) is greater than that of women (Figure 1B). As can also be seen in Figure 1E,F, the apparent volumes in the whole cell (Figure 1E) and nuclear (Figure 1F) levels are significantly (*p* < 0.0001) higher in young men than in young women. Thus, an essential difference at the morphological level exists between men’s and women’s VSMCs.

In a second series of protocols, we wanted to verify whether hyperinsulinemia induced VSMC hypertrophy differently from men and women. In this series of experiments, we tested the effect of long-term pretreatment (48 h) of hyperinsulinemia. Figure 1A–D shows typical experiments, and Figure 1E,F summarizes the results. Figure 1 shows treatment with a high concentration of insulin-induced increase in cells (Figure 1A–E) and nuclear (Figure 1A–C,F). This effect was highly significant (*p* < 0.0001) (Figure 1E,F). In addition, as shown in Figure 1A–F, hyperinsulinemia did not change the difference in volume between VSMCs from men and women.

### 3.2. Effect of 48 h Treatment of Hyperinsulinemia (80 μU/mL) on the Intracellular Ca^2+^ Levels of VSMCs from Men and Women

**Figure 2:**
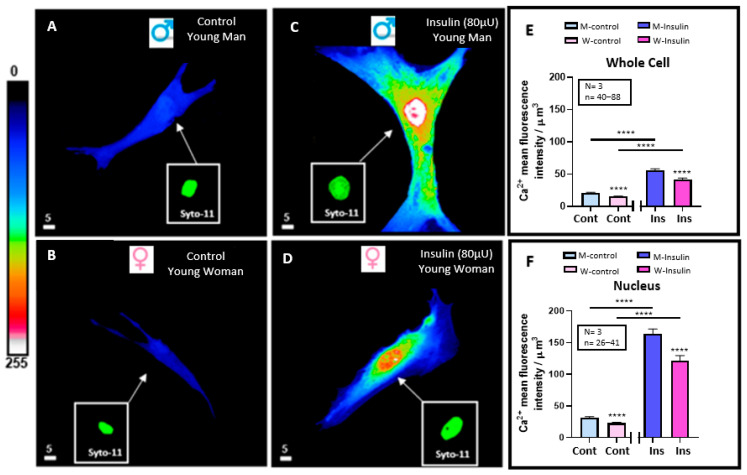
Effect of 48 h treatment of hyperinsulinemia (80 μU/mL) on intracellular Ca^2+^ levels of VSMCs from men and women. Examples of a typical quantitative 3D top-view image of VSMCs: in absence of hyperinsulinemia in VSMCs from men (**A**) and women (**B**) and with hyperinsulinemia in VSMCs from men (**C**) and women (**D**) with statistical significance of their whole cell (**E**) and nuclear (**F**) Ca^2+^ levels. Panels (**E**,**F**) show a significant increase in intracellular Ca^2+^ levels of VSMCs from both men and women induced by 48 h of hyperinsulinemia. Inset panels (green staining) represent nuclear labeling of the cells observed in panels (**A**–**D**) with Syto-11. Pseudocolor scale represents Ca^2+^ fluorescence intensity ranging from 0 (no fluorescence) to 255 (maximum fluorescence). White scale bar is μm. Values are expressed by μm^3^ and represented as mean ± standard error of the mean. N indicates number of donors, while n refers to number of cells. **** *p* < 0.0001. M: men, W: women, Cont: control, Ins: insulin.

In another series of experiments, we wanted to verify whether hyperinsulinemia-induced VSMC hypertrophy is associated with increased whole and nuclear VSMC Ca^2+^ differently from men and women. Figure 2A–D shows typical examples, and Figure 2E,F summarizes the results. As seen in Figure 2A,B, the basal resting level of both whole cells and their nuclei is significantly higher (*p* < 0.0001) in men than in women (Figure 2E,F). Figure 2 also shows treatment with a high concentration of insulin-induced increase in whole cells (Figure 2A–E) and nuclear-free Ca^2+^ (Figure 2A–D,F). This effect was highly significant (*p* < 0.0001) (Figure 2E,F). In addition, as shown in Figure 2A–F, hyperinsulinemia did not change the difference in volume, and its associated difference increased the level of whole cell and nuclear Ca^2+^ levels between VSMCs from men and women.

### 3.3. Effect of 48 h Treatment of Hyperinsulinemia (80 μU/mL) on the Intracellular ROS^+^ Levels of VSMCs from Men and Women

**Figure 3:**
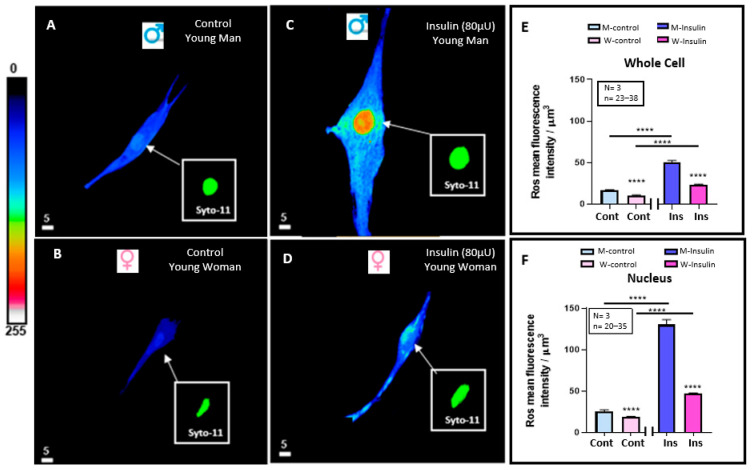
Effect of 48 h treatment of hyperinsulinemia (80 μU/mL) on the intracellular ROS levels of VSMCs from men and women. Examples of a typical quantitative 3D top-view image of VSMCs: in absence of hyperinsulinemia in VSMCs from men (**A**) and women (**B**) and with hyperinsulinemia in VSMCs from men (**C**) and women (**D**) with statistical significance of their whole cell (**E**) and nuclear (**F**) ROS levels. Panels (**E**,**F**) show a significant increase in intracellular ROS levels of VSMCs from both men and women induced by 48 h of hyperinsulinemia. Inset panels (green staining) represent nuclear labeling of the cells observed in panels (**A**–**D**) with Syto-11. Pseudocolor scale represents ROS fluorescence intensity ranging from 0 (no fluorescence) to 255 (maximum fluorescence). White scale bar is in μm. Values are expressed by μm^3^ and represented as mean ± standard error of the mean. N indicates number of donors, while n refers to number of cells. **** *p* < 0.0001. M: men, W: women, Cont: control, Ins: insulin.

In this series of experiments, we wanted to verify whether the basal resting level of ROS in VSMCs is different in men and women and if hyperinsulinemia-induced VSMC hypertrophy is associated with increased whole and nuclear VSMC ROS and sex-dependent. Figure 3A–D shows typical examples, and Figure 3E,F summarizes the results. Figure 3A,B showed the basal resting level of both whole cells and their nuclei is significantly higher (*p* < 0.0001) in men than in women (Figure 3E,F). In addition, Figure 3 shows treatment with a high concentration of insulin-induced increase in whole cells (Figure 3A–E) and nuclear-free ROS (Figure 3A–D,F). This effect was highly significant (*p* < 0.0001) (Figure 3E,F). Furthermore, as shown in Figure 3A–F, hyperinsulinemia did not change the difference in volume, and its associated difference increased the level of whole cell and nuclear ROS levels between VSMCs from men and women.

### 3.4. Effect of 48 h Treatment of Hyperinsulinemia (80 μU/mL) on the Intracellular Glutathione Levels of VSMCs from Men and Women

**Figure 4:**
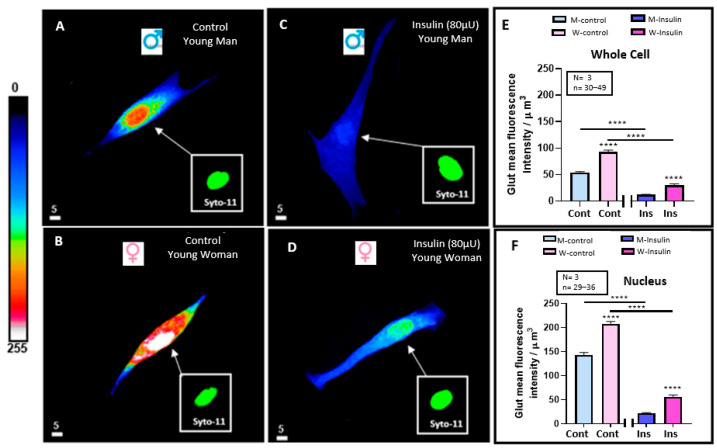
Effect of 48 h treatment of hyperinsulinemia (80 μU/mL) on the intracellular glutathione levels of VSMCs from men and women. Examples of a typical quantitative 3D top-view image of VSMCs: in absence of hyperinsulinemia in VSMCs from men (**A**) and women (**B**) and with hyperinsulinemia in VSMCs from men (**C**) and women (**D**) with statistical significance of their whole cell (**E**) and nuclear (**F**) glutathione levels. Panels (**E**,**F**) show a significant decrease in intracellular glutathione levels of VSMCs from both men and women induced by 48 h of hyperinsulinemia. Inset panels (green staining) represent nuclear labeling of cells observed in panels (**A**–**D**) with Syto-11. Pseudocolor scale represents glutathione fluorescence intensity ranging from 0 (no fluorescence) to 255 (maximum fluorescence). White scale bar is in μm. Values are expressed by μm^3^ and represented as mean ± standard error of mean. N indicates number of donors, while n refers to number of cells. **** *p* < 0.0001. M: men, W: women, Cont: control, Ins: insulin.

In another series of experiments, we wanted to verify whether the basal resting level of the endogenous anti-ROS level glutathione of VSMCs differs in men and women. Figure 4A,B shows typical 3D images, and Figure 4E,F shows the statistical summaries. As seen in Figure 4A,B, the basal resting level of both whole cells and their nuclei is significantly higher (*p* < 0.0001) in women than in men (Figure 4E,F).

In a second series of experiments, we verified whether hyperinsulinemia-induced VSMC hypertrophy is associated with a decrease in whole cell and nuclear glutathione levels and if it is different in men and women. Figure 4A,B shows typical examples, and Figure 4E,F summarizes the results. Figure 4C,D demonstrates that treatment with a high concentration of insulin-induced decrease in whole cells (Figure 4A–E) and nuclear-free glutathione (Figure 4A–D,F). This effect was highly significant (*p* < 0.0001) (Figure 4E,F). In addition, as shown in Figure 4A–F, hyperinsulinemia did not change the difference in volume, and its associated difference increased the level of whole cell and nuclear glutathione levels between VSMCs from men and women.

### 3.5. Effect of 48 h Treatment of Hyperinsulinemia (80 μU/mL) in the Absence and Presence of Testosterone (10^−9^ M) or Estrogen (10^−10^) M on the Volume of VSMCs from Men and Women

**Figure 5:**
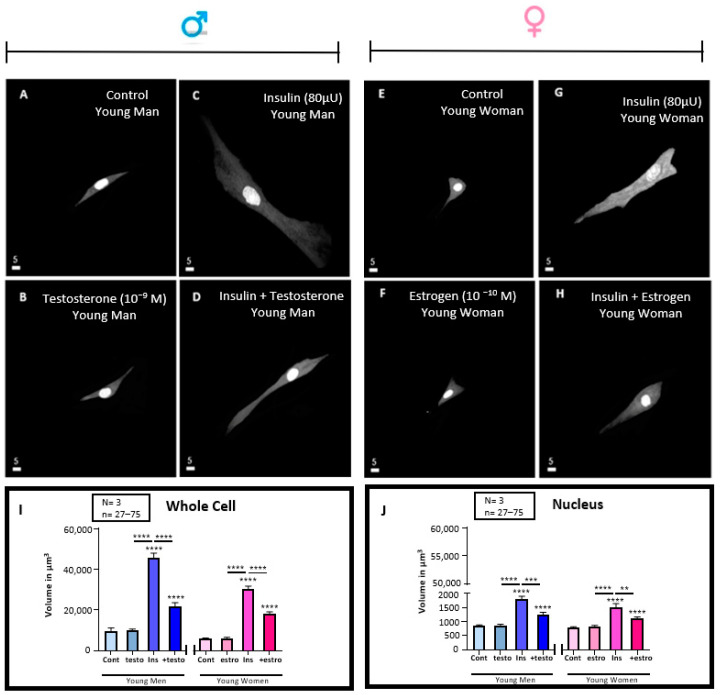
Effect of 48 h treatment of hyperinsulinemia (80 μU/mL) in absence and presence of testosterone (10^−9^ M) or estrogen (10^−10^) M on volume of VSMCs from men and women. Histograms represent whole cell (**I**) and nuclear (**J**) volume of VSMCs from men and women under normal and hyperinsulinemia conditions in the absence and presence of a 48 h treatment with 10^−9^ M of testosterone and 10^−10^ M of estrogen. Panels (**A**,**E**) are typical examples of a real quantitative 3D top view from men’s (**A**) and women’s (**E**) VSMCs volume under normal conditions. Treatment with 10^−9^ M of testosterone and 10^−10^ M of estrogen did not significantly change cellular and nuclear volume of VSMCs from men (**B**) and women (**F**) (panels **I**,**J**). However, hyperinsulinemia induced a significant increase in cellular and nuclear volume of VSMCs from men (**C**) compared to women (**G**) (panels **I**,**J**). Pretreatment with 10^−9^ M of testosterone and 10^−10^ M of estrogen partially but significantly prevented increase in cell and nuclear volume induced by hyperinsulinemia in VSMCs from men (**D**) and women (**H**) (panels **I**,**J**). However, addition of sex hormones did not significantly (*p* < 0.0001) abolish the sexes’ differences of VSMCs regarding their response to chronic high insulin levels (**I**,**J**). Nuclei are labelled with syto-11. White scale bar is in μm. Values are expressed in μm^3^ and represented as mean ± standard error of the mean. N indicates number of donors, while n refers to number of cells. **** *p* < 0.0001, ** *p* < 0.01. Testo: testosterone, Estro: estrogen, Cont: control, Ins: Insulin.

In a series of experiments, we wanted to verify whether treatment for 48 h with testosterone and estrogen affects (in the absence of insulin), the volume of VSMCs from men and women. Figure 5A,B shows an example of 3D images of VSMCs from men and women. Figure 5I–J summarizes the results in whole cell volume (Figure 5I) and their nuclei (Figure 5J). These figures show that testosterone and estrogen did not affect VSMC volume.

In another series of experiments, we verified whether pretreatment with testosterone in VSMCs from men and estrogen pretreatment from women’s cells prevent the hyperinsulinemia-induced increase of cell volume of men and women in a sex-dependent manner. Figure 5C,D,G,H show typical examples of 3D cell images, and Figure 5I,J show and summarize the results in whole cells and nuclear volumes. As seen in Figure 5D,H, pretreatment with testosterone in VSMCs from men, as well estrogen in cells of women, partially (but significantly) prevented the hyperinsulinemia-induced increase of whole cell (*p* < 0.0001) and nuclear (*p* < 0.01) volumes (I, J). In addition, as shown in Figure 5I,J, hyperinsulinemia in the presence of sexual hormones did not change the difference in volume between VSMCs from men and women.

### 3.6. Effect of 48 h Treatment of Hyperinsulinemia (80 μU/mL) in the Absence and Presence of Testosterone (10^−9^ M) or Estrogen (10^−10^) M on the Whole Cells and Their Nuclear Ca^2+^ Levels of VSMCs from Men and Women

**Figure 6:**
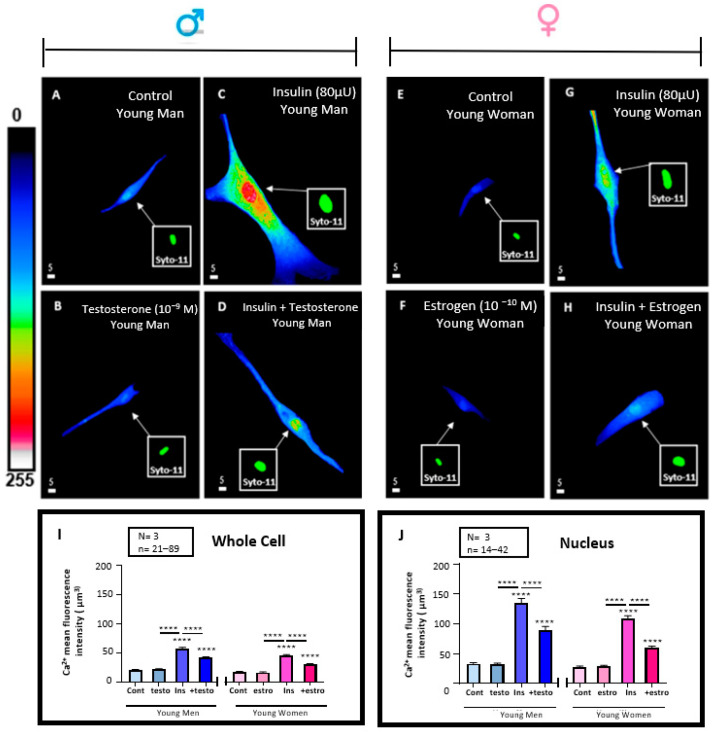
Effect of 48 h treatment of hyperinsulinemia (80 μU/mL) in absence and presence of testosterone (10^−9^ M) or estrogen (10^−10^) M on Ca^2+^ levels of VSMCs from men and women. Histograms represent whole cell (**I**) and nuclear (**J**) Ca^2+^ levels of VSMCs from men and women under normal and hyperinsulinemia conditions in absence and presence of a 48 h treatment with 10^−9^ M of testosterone and 10^−10^ M of estrogen. Panels (**A**,**E**) are typical examples of a real quantitative 3D top view of intracellular Ca^2+^ levels from men’s (**A**) and women’s (**E**) VSMCs under normal conditions. Treatment with 10^−9^ M of testosterone and 10^−10^ M of estrogen did not induce a significant change in cellular and nuclear Ca^2+^ levels in VSMCs from men (**B**,**I**,**J**) and women (**F**,**I**,**J**). However, hyperinsulinemia significantly increased cellular and nuclear Ca^2+^ levels of VSMCs from men (**C**,**I**,**J**) compared to women (**G**,**I**,**J**) VSMCs. Pretreatment with 10^−9^ M of testosterone and 10^−10^ M of estrogen partially but significantly prevented increase in cell and nuclear Ca^2+^ levels induced by hyperinsulinemia in VSMCs from men (**D**,**I**,**J**) and women (**H**,**I**,**J**). However, addition of sex hormones did not significantly (*p* < 0.0001) abolish the sexes’ differences of VSMCs regarding their response to chronic high insulin levels. Inset panels (green staining) represent nuclear labeling of the cells observed in panels (**A**–**H**) with Syto-11. Pseudocolor scale represents Ca^2+^ fluorescence intensity ranging from 0 (no fluorescence) to 255 (maximum fluorescence). White scale bar is in μm. Values are expressed by μm^3^ and represented as mean ± standard error of the mean. N indicates number of donors, while n refers to number of cells. **** *p* < 0.0001. Testo: testosterone, Estro: estrogen, Cont: control, Ins: insulin.

In this series of experiments, we wanted to verify whether treatment for 48 h with testosterone in men and with estrogen in women VSMCs affects basal resting levels (in the absence of insulin). Figure 6A,B shows an example of 3D images of VSMCs from men and Figure 6E,F of women. Figure 6I,J summarizes the results in whole cell volume (Figure 6I) and their nuclei (Figure 6J) of VSMC from men and women. These figures show that testosterone and estrogen did not affect VSMCs’ free intracellular Ca^2+^ without hyperinsulinemia.

In another series of experiments, we verified whether pretreatment with testosterone in men and estrogen pretreatment in women VSMCs prevent the hyperinsulinemia-induced increase of Ca^2+^ in whole cells and their nuclei in a sex-dependent manner. Figure 6C,D,G,H show typical examples of 3D cell images, and Figure 6I,J show and summarize the results in whole cells and nuclear volumes. As seen in Figure 6C,D, pretreatment with testosterone in VSMCs from men, as well estrogen (Figure 6G,H) in women, partially (but significantly) prevented the hyperinsulinemia-induced increase of whole cell (*p* < 0.0001) and nuclear (*p* < 0.0001) (Figure 6I,J) Ca^2+^ levels. In addition, as shown in Figure 6I,J, hyperinsulinemia in the presence of sexual hormones did not change the difference in intracellular Ca^2+^ between VSMCs from men and women.

## 4. Discussion and Conclusions

In a healthy individual, the physiological concentration of insulin typically ranges from 15 to 45 µU/mL. However, in certain conditions, such as hyperinsulinemia and T2D, the pathological concentration of insulin can rise between 80 and 100 µU/mL [13,28,29]. For many years, hyperinsulinemia was believed to result from insulin resistance [4,13]. However, it has recently been suggested that the ‘modern’ western diet is the cause of hyperinsulinemia preceding the development of insulin resistance and T2D in individuals [4]. Chronic and continuous exposure to foods from the ‘modern’ diet may lead to insulin hypersecretion from the pancreatic beta cells, thus resulting in hyperinsulinemia [4,30]. Therefore, a new hypothesis was proposed that chronic hyperinsulinemia not only precedes but causes insulin resistance, eventually leading to T2D [4]. Hyperinsulinemia leads to more insulin binding to its receptors on target tissues [31]. This suggests that hyperinsulinemia may overstimulate insulin receptors, resulting in insulin resistance, which ultimately leads to the development of T2D [31].

Furthermore, hyperinsulinemia not only precedes insulin resistance and T2D but also contributes to the development of obesity [2,3,18]. Under normal physiological conditions, the vascular endothelium and VSMCs contain abundant insulin receptors critical in maintaining vascular health and regulating blood vessel tone [32]. However, when a pathology like chronic hyperinsulinemia occurs (in presence or absence of hyperglycemia) it can lead to insulin resistance as there is an increase in insulin signaling, ultimately leading to vascular diseases such as hypertension and atherosclerosis [3,32]. The endothelium and contractile VSMCs of the tunica media are constantly exposed to circulating substances such as high levels of insulin and lipids [8]. These factors promote morphological and intracellular remodeling of blood vessels [8]. Hyperinsulinemia is among the pathological risk and comorbidities factors leading to the development of SAH [20,33]. Although the exact mechanisms by which hyperinsulinemia contributes to SAH are not fully understood, they are thought to involve ionic and morphological remodeling induced by elevated blood insulin levels [20,33]. In addition, recent epidemiological work suggests that age and sex may contribute to SAH [34] as well as in conditions of andropause in aging men and menopause in aging women [2].

Our results showed that there are intrinsic properties of VSMCs from young men which are different from that of VSMCs from young women. There is a fundamental inherent sex difference between the VSMCs from young men and women in size, intracellular Ca^2+^, ROS, and endogenous anti-ROS levels. Such a difference would affect the blood pressure of both sexes differently. The increase in the size of the VSMCs would increase the thickness of the tunica media, thus reducing the lumen of the blood vessel [7] and promoting both SAH and atherosclerosis. The small size of VSMCs from young women compared to young men may decrease the tunica media size, thus increasing the lumen size and blood flow [7]. In addition, the low level of intracellular Ca^2+^ in young women VSMCs compared to young men may partly explain the lower blood pressure in young women compared to young men [17,18,19,20]. This may explain why women develop less hypertension and atherosclerosis when compared to men [35]. Whether these differences in young men and women persist in menopausal older women and older men needs to be explored.

Our results also showed that intrinsic differences in the levels of ROS and glutathione exist between young men and women. The lower ROS and the higher glutathione in young women compared to young men VSMCs may better protect young women’s tunica media compared to young men. This may also explain, at least in part, the well-known lower incidence of SAH and atherosclerosis in young women when compared to young men [35,36]. The more significant oxidative stress and lower glutathione in VSMCs from healthy young men when compared to young women were also reported in clinical trials using plasma samples [37,38]. More work should be done to explore the VSMC’s intrinsic differences in cell physiology, pharmacology, and signaling between young men and women as well as in aging individuals.

Our results also showed that chronic hyperinsulinemia-induced morphological remodeling in both VSMCs from young men and women was associated with increased cell and nuclear volume. The percentage of increase of the volume by chronic hyperinsulinemia is nearly similar in men and women. However, the increase of the nuclear volume (hypertrophic marker) by hyperinsulinemia suggests that the increase in cell volume is hypertrophy [27]. These results suggest that chronic hyperinsulinemia does not affect the fundamental differences between VSMCs from young men and women. More work should be done in order to confirm these findings.

Our results also showed that chronic hyperinsulinemia-induced increase in cytosolic and nuclear Ca^2+^ in both VSMCs from young men and women. However, it did not affect the lower level of intracellular Ca^2+^ in young women when compared to young men. Such an essential increase of cytosolic and nuclear Ca^2+^ may highly promote the development of SAH. There is no doubt that the very high increase of nuclear Ca^2+^ by chronic hyperinsulinemia may also promote the activation of Ca^2+^-dependent mechanisms at both cytosolic and nuclear levels [9,39]. The mechanisms responsible for the increase of intracellular Ca^2+^ (Ca^2+^ channels, exchangers, and ryanodine receptors) as well as direct and indirect Ca^2+^-dependent hypertrophic transcriptional factors (CREB, CaMKII, and calcineurin) remain to be determined [9,39].

As for Ca^2+,^ our results showed that the increase of VSMC volume and intracellular Ca^2+^ by chronic hyperinsulinemia is associated with an increase in ROS level and, more importantly, at the nuclear level. The relative percentage increase of ROS is more critical in VSMCs from young men than women. These results suggest that hyperinsulinemia contributes to higher ROS generation in VSMCs from young men when compared to young women. As such, a significant increase of oxidative stress by chronic hyperinsulinemia in young men compared to young women is similar to what was reported in T2D [40]. Our results show that chronic hyperinsulinemia decreased cytosolic and nuclear glutathione levels. This is expected since the increase of ROS in hyperinsulinemia is chelated by glutathione. As for volume, Ca^2+,^ and ROS, the intrinsic difference in glutathione levels between VSMCs from young men and women was not affected. These results suggest that antioxidants could be a good tool for preventing or blocking chronic hyperinsulinemia-induced remodeling of VSMCs from young men and women [38]. This should be verified in the future.

Finally, our results showed that the sex hormones of young men and women did not affect the intrinsic property of VSMCs from both sexes. This was demonstrated by the absence of the effect of sex hormones on cellular volume and intracellular Ca^2+^. Thus, the inherent properties of VSMCs from young men and women is independent of their sex hormones. Furthermore, our results showed that sex hormones did not completely (partially) prevent hyperinsulinemia-induced increase in cell volume and intracellular Ca^2+^. These results suggest that chronic hyperinsulinemia-induced remodeling of VSMCs from young men and women is relatively independent of their sex hormones. Whether the level of ROS and glutathione is affected by treatment with sex hormones should be verified in the future and the mechanism should be determined. It is important to mention that the in vivo situation of sex hormones is very complex since both men and women have androgens and estrogens but at different concentrations. Therefore, it is important to take these aspects in consideration in future studies.

In conclusion, our results demonstrate that intrinsic properties exist that differentiate VSMCs from young men from women. This partly explains the differences in the development of cardiovascular diseases in young men and women. In addition, chronic hyperinsulinemia induced similar remodeling in VSMCs from both sexes. Furthermore, since sex hormones were reported to have antioxidant effects [23], it is possible that treatment with an antioxidant may constitute a promising treatment for hyperinsulinemia-induced SAH and its related development of T2D and atherosclerosis. Since in general, it is difficult (even impossible) to determine in vivo the remodeling and the mechanism responsible for hyperinsulinemia in human VSMCs, our results demonstrate that this is possible to be done in vitro using cells isolated from young men and women organ donors. Thus, our work demonstrates that in vitro use of VSMCs from human organ donors constitute a good model to study hyperinsulinemia and its related vascular disease. More work in vitro should be done to explore the mechanisms responsible for chronic hyperinsulinemia-induced remodeling of VSMCs from young men and women as well as in aging humans.

## Data Availability

Data are contained within the article.
